# Bone marrow-derived cells can acquire renal stem cells properties and ameliorate ischemia-reperfusion induced acute renal injury

**DOI:** 10.1186/1471-2369-13-105

**Published:** 2012-09-10

**Authors:** Xiaohua Jia, Xiaoqiang Xie, Guowei Feng, He Lű, Qinjun Zhao, Yongzhe Che, Yizhou Zheng, Zhongchao Han, Yong Xu, Zongjin Li, Deling Kong

**Affiliations:** 1 State Key Laboratory of Medicinal Chemical Biology, Key Laboratory of Bioactive Materials, Ministry of Education, Nankai University, Tianjin 300071, China; 2 School of Medicine, Nankai University, Tianjin 300071, China; 3 Department of Urology, Second Hospital of Tianjin Medical University, Tianjin Institute of Urology, Tianjin 300211, China; 4 College of Basic Medicine, Yanbian University, Yanji, Jilin 133002, China; 5 State Key Lab of Experimental Hematology, Institute of Hematology & Hospital of Blood Diseases, Chinese Academy of Medical Sciences, Tianjin 300020, China

**Keywords:** Bone marrow transplantation, Trans-differentiation, Renal stem cell, Acute kidney injury, G-CSF, Mobilization

## Abstract

**Background:**

Bone marrow (BM) stem cells have been reported to contribute to tissue repair after kidney injury model. However, there is no direct evidence so far that BM cells can trans-differentiate into renal stem cells.

**Methods:**

To investigate whether BM stem cells contribute to repopulate the renal stem cell pool, we transplanted BM cells from transgenic mice, expressing enhanced green fluorescent protein (EGFP) into wild-type irradiated recipients. Following hematological reconstitution and ischemia-reperfusion (I/R), Sca-1 and c-Kit positive renal stem cells in kidney were evaluated by immunostaining and flow cytometry analysis. Moreover, granulocyte colony stimulating factor (G-CSF) was administrated to further explore if G-CSF can mobilize BM cells and enhance trans-differentiation efficiency of BM cells into renal stem cells.

**Results:**

BM-derived cells can contribute to the Sca-1^+^ or c-Kit^+^ renal progenitor cells population, although most renal stem cells came from indigenous cells. Furthermore, G-CSF administration nearly doubled the frequency of Sca-1+ BM-derived renal stem cells and increased capillary density of I/R injured kidneys.

**Conclusions:**

These findings indicate that BM derived stem cells can give rise to cells that share properties of renal resident stem cell. Moreover, G-CSF mobilization can enhance this effect.

## Background

Chronic kidney disease (CKD) and end-stage renal disease (ESRD) are associated with considerable morbidity and mortality. Current treatments fail to cure CKD and can’t halt progression of CKD to ESRD [1]. Despite advances in the understanding of glomerular and tubular injury and regeneration, therapeutic advances have been limited because of the organ shortage for renal transplantation and the complexity of kidney. Stem cell-based therapy is a new strategy in the treatment of acute kidney injury and has potentially more value than single-agent drug therapy due to the highly versatile response of cells to their environment [2]. The potential cellular sources for kidney regeneration include renal resident stem cells and extra-renal stem cells. A number of recent studies have confirmed the presence of cells bearing stem cell markers such as Sca-1, c-Kit, and CD133 in the kidney [3-10]. These cells can differentiate, proliferate, and eventually reline denuded tubules, restoring the structural and functional integrity of the kidney. However, the homeostasis of renal stem cells in kidney is still under investigation.

Differentiation of bone marrow (BM) derived-cells into cells of non-haematopoietic origin has been described in several *in vivo* studies and gave rise to the thought that the BM-derived cells population could be involved in tissue turnover and regeneration, including kidney [2,11-13]. It has been hypothesized that the stem cell repertoire of adult tissues generally consists of a small number of self-replenishing cells that differentiate from primate bone marrow cells [14]. The movement of stem cells is critical for homeostasis and repair in adulthood.

Identification of stem cells in kidney tissues is important for therapeutic applications and for understanding developmental processes and tissue homeostasis. Previous research revealed that the BM-derived hematopoietic stem cells (HSCs) can reside in kidney and differentiate into mature cells [15]. Furthermore, the turning over of BM derived stem cells into renal stem cells has not been investigated so far. In this study, we sought to address the plasticity of BM stem cells in trans-differentiating into renal stem cells and BM derived stem cells in kidney regeneration after acute kidney injury (AKI), as well as the fortified effects of granulocyte colony-stimulating factor (G-CSF) in mobilizing bone marrow stem cells trans-differentiate into renal stem cells.

## Methods

### Isolation and transplantation of bone marrow cells

Animal protocols were approved by the Nankai University Animal Care and Use Committee. Mice were anesthetized with inhaled isoflurane (2% to 3%). 8 to 10-week-old female C57BL/6 J mice (The Laboratory Animal Center of The Academy of Military Medical Sciences, Beijing, China) (n = 30 per group) were irradiated with 9.5 Gy of γ-irradiation in 2 divided doses, 2 hours apart, on the day of surgery. Bone marrow cells were isolated from the femur and tibia of 8 to 10-week-old female C57BL/6 J-TgN mice (The Laboratory Animal Center of The Academy of Military Medical Sciences, Beijing, China), transgenically expressing the chicken β-actin-EGFP gene, by flushing with Iscove’s minimal essential medium (IMEM). For bone marrow transplantation (BMT), wild-type irradiated mice were injected with 0.2 ml PBS with or without 2.0 × 10^5^ BM mononuclear cells via tail veins at 2 hours after irradiation. Mice were kept in a specific pathogen free facility and drinking water containing enrofloxacin (0.15 mg/ml) and amoxicillin (1 mg/ml) were given for 4 weeks to prevent infection.

### Acute kidney ischemia/reperfusion experiments

Acute left kidney ischemia/reperfusion was carried out at 5-week after BMT. Mice were anesthetized by intraperitoneal injection with 300 mg/kg chloraldurat. Animals were placed on a heating pad to maintain a constant temperature and monitored with a rectal thermometer. A midline abdominal incision was made, and left kidneys were exposed. The left renal artery was separated from the vein and clamped for 30 min followed by clamp release to allow reperfusion. Throughout ischemic period, evidence of clamping was confirmed by visualizing dark color of ischemic kidneys. After clamp removal, adequate restoration of blood flow was checked before abdominal closure. Sham-operated animals underwent anesthesia, laparotomy, and renal pedicle dissection only.

### Mobilization of bone marrow stem cells following ischemic injury

For HSCs mobilization, mice received a subcutaneous injection of 200 μg/kg recombinant G-CSF daily for 8 days from 5 before induction of ischemia. Control mice received an injection of saline (n = 15 per group).

### Flow cytometry analysis

To measure stem cells mobilization, flow cytometry analyses (FACScan flow cytometer, Becton Dickinson) were performed on day 9 and 33 after administration of G-CSF. The peripheral blood was stained with Alexa Fluor® 647-conjugated rat anti-mouse CD34, APC-labeled anti-mouse c-Kit and CD45, and PE-labeled rat anti-mouse Sca-1, Flk-1 and rat anti-mouse CD29, (all from BD Pharmingen). 5 weeks after BMT, bone marrow engraftment efficiency in recipients was determined by analyzing GFP expression of peripheral blood.

### Isolation and characterization of renal progenitor cells after BMT

Nine weeks after BMT, the intact kidney tissue was minced and added to 10 ml of a 4 mg/ml solution of dispase (sigma-Aldrich) in DMEM (Invitrogen). The minced tissue and media were transferred to a 50-ml Erlenmeyer flask and incubated for 1 h at 37°C. Following the incubation, the tissue was filtered through 40 μm nylon cell strainer (BD Pharmingen) to remove cell segments [7]. Kidney cell suspensions were washed twice in DMEM and stained with Sca-1, c-Kit, Flk-1, CD29, CD34 and CD45 for FACS analysis.

### Histology and immunohistochemistry

Four, 8 and 48 weeks after I/R, mice were euthanized and the kidneys were thoroughly perfused with saline to remove blood from the vascular beds. The specimens of kidney were embedded into paraffin or OCT compound (Miles Scientific), then sectioned to 5 μm slides and processed for hematoxylin-eosin, Masson staining and immunostaining. To track BM-derived cells in kidneys, rabbit anti-c-Kit antibody (Santa Cruz), mouse monoclonal smooth muscle actin (α-SMA, Boster Co., China), monoclonal anti-mouse Sca-1 (Cedarlane), and rat anti-mouse CD45, CD29, CD105 (all from BD Pharmingen) were used. Alexa Fluor 594 and Alexa Fluor 488-conjugated secondary antibodies (Invitrogen) were applied appropriately. DAPI was used for nuclear counterstaining. To detect vascular density in the infarct area, a rat anti-mouse CD31 antibody was used. For immunohistochemistry, the endogenous peroxides were blocked using 3% H_2_O_2_ in PBS at room temperature for 10 minutes. After being blocked with 10% goat serum in PBS, the sections were stained with primary antibody (mouse monoclonal anti-GFP, Abcam 1:500) overnight at 4°C, biotinylated secondary antibody for 45 minutes at 37°C, and diaminobenzidine reagent (Vector Laboratories) for 4 minutes. The numbers of BM-derived renal stem cells and capillary vessels were counted by a blinded investigator (LH) in 10 randomly selected high-power fields (HPF) using a fluorescence microscope (×400) [12]. The blood vessel density was expressed as capillaries/HPF (×400). The numbers of BM-derived renal stem cells were counted as GFP^+^Sca-1^+^ cells/HPF and GFP^+^c-Kit^+^ cells/HPF (×400).

### Statistical analysis

All data are expressed as mean values ± SEM. One-way analysis of variance was employed for comparing differences between groups. Least significant difference (equal variances) and Dunnett’s T3 (non-equal variances) post hoc tests were used for testing the differences between groups. All tests were two-tailed, and significance was accepted at *P <* 0.05.

## Results

### Hematopoietic reconstruction and engraftment of GFP positive cells into damaged kidney

**Figure 1:**
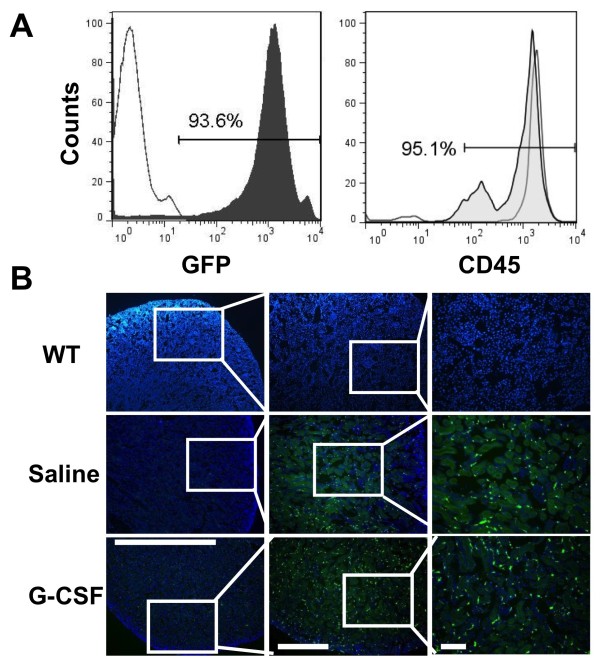
**Engraftment of BM derived cells into I/R-injured kidneys.** (**A**) Confirmation of chimerism in mice transplanted with bone marrow from GFP mice. Representative peripheral blood from wild-type (WT) mice and chimeric BMT mice 5 weeks after BMT were analysised by FACS. CD45 expression in recipient mice was also evaluated at the same time point. (**B**) BM-derived GFP positive cells within recipient kidneys. No GFP^+^ cell was observed in WT mice, while donor derived GFP^+^ cells were widely infiltrated in saline-treated mice. G-CSF administration mobilized more BM-derived cells to the injured kidney compared to the saline-treated mice. Nuclei were stained with DAPI (blue). Scale bars = 50 μm.

Five weeks after BMT, hematopoietic reconstruction of recipient mice were confirmed by robust expression of GFP (83.0 ± 4.2%) and CD45 (93.4 ± 5.2%) in peripheral blood by FACS analysis (Figure 1A). Four weeks after I/R injury, kidneys were harvested and the engraftment of BM-derived GFP^+^ cells into damaged kidneys was evaluated by histology. There were widespread GFP^+^ cells infiltrated in the injured kidney of saline-treated group, implicating the migration and engraftment of BM derived stem cells into kidneys. What is intriguing, much more GFP^+^ cells were observed in the G-CSF administered mice compared to the saline-treated ones. But no GFP^+^ cells were detected in the damaged kidney of wild type (WT) mice (Figure 1B). Furthermore, GFP expression in kidney from chimeric mice that underwent I/R injury was confirmed by immunohistochemical staining. Additional file 1: Figure S1 showed that GFP^+^ cells localized in the glomeruli and interstitium in consecutive sections of the injured kidney from the mice sacrificed 6 months after ischemic injury.

### Bone marrow-derived cells can acquire renal stem cells properties

**Figure 2:**
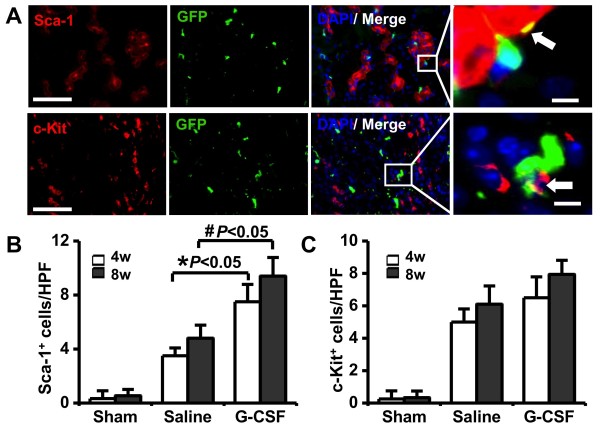
**BM derived stem cells can acquire renal stem cells characteristics.** (**A**) Renal stem cells positive for GFP and Sca-1 or c-Kit were showed in merge (arrowhead). Nuclei were stained with DAPI (blue). Scale bars = 50 μm (left) and 5 μm (right), respectively. (**B**) Quantitative analysis of renal stem cells revealed that G-CSF administration nearly doubled the frequency of Sca-1 expressing BM-derived renal stem cells. *P < 0.05 vs. 4w Saline group; ^#^*P <* 0.05 vs. 8w Saline group. (**C**) Quantitative analysis of c-Kit positive renal progenitor cells. HPF: high-power field, (×400).

BM derived stem cells differentiation and acquisition of renal fate involved the engraftment of the donor cells within the host kidney. 4 weeks after injury, transplanted BM derived stem cells were seeded within renal tubules and were integrated structurally with resident cells. However, most BM derived stem cells were CD45-negative (data not shown). The presence of GFP was used to distinguish resident from injected CD45-positive cells. Immunostaining demonstrated that BM-derived cells contributed to the Sca-1^+^ or c-Kit^+^ renal stem cells, although most renal stem cells came from indigenous cells (Figure 2A). Some Sca-1^+^ or c-Kit^+^ cells were detected in the engrafted BM derived stem cells (Figure 2A), and more Sca-1^+^/GFP^+^ or c-Kit^+^/GFP^+^ renal stem cells in the ischemic kidneys (Figure 2B-C), which suggesting that the renal microenvironment may change the fate of BM derived stem cells, and BM derived stem cells home to the kidney where they lose the hematopoietic phenotype and acquire renal stem cells lineage.

### G-CSF can mobilize BM-derived stem cells and enhance differentiation of BM cells into renal stem cells

**Figure 3:**
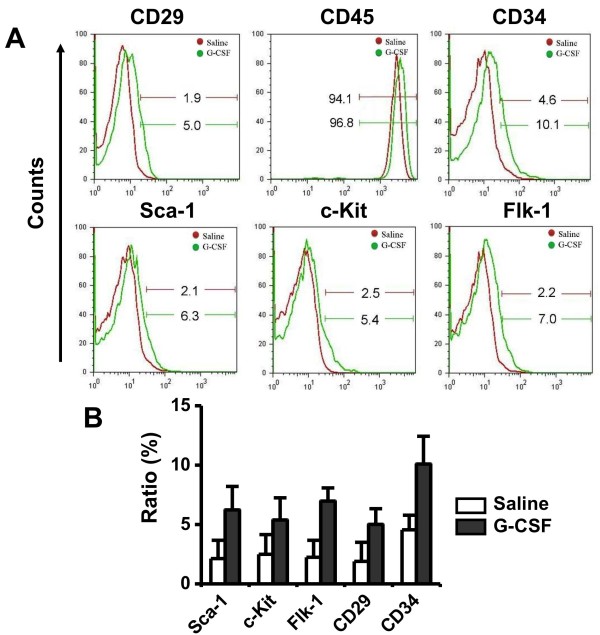
**G-CSF increased the mobilization of BM stem cells into peripheral blood.** (**A**) FACS analysis of peripheral blood, saline (red line) or G-CSF (green line). (**B**) Quantitative analysis of cell markers examined by FACS. Comparing with saline group, G-CSF group expressed higher stem cell markers such as Sca-1, c-Kit, Flk-1, CD29 and CD34.

To determine whether G-CSF induced stem cells mobilization would affect renal recovery, FACS and immunohistology analysis were carried out. Analysis of peripheral blood revealed that one day after last injection of G-CSF, all of the stem cell markers were increased in peripheral blood mobilized with G-CSF as compared with control group (Figure 3). There was a kind of respectively more than 2-fold increase in the total number of CD34^+^, Sca-1^+^ or c-Kit^+^ cells in cytokine-treated animals compared with controls. However, G-CSF treatment did not lead to significantly increased numbers of circulating CD45^+^ cells compared with controls (91.1 ± 5.3% versus 96.8 ± 6.5%, *P* > 0.05).

**Figure 4:**
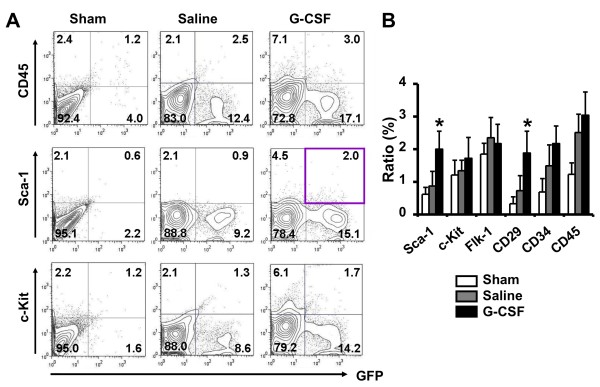
**G-CSF increased the differentiation efficiency of BM stem cells into renal stem cells.** (**A**) 4 weeks after I/R injury, FACS analysis BM derived GFP^+^ cells in kidney. The Sca-1^+^/GFP^+^ cells were significantly up-regulated in G-CSF treated groups (purple box). (**B**) Evaluation of GFP positive stem cells in kidney cells by FACS. *P < 0.05 vs. 4w Saline group.

Furthermore, 4 weeks after I/R injury, the flow cytometry examination of kidney cells revealed the number of BM-derived GFP^+^ cells and the apparent populations of stem cells in the adult kidneys. G-CSF promoted more stem cell engraftment in damaged kidneys comparing with saline treated (Figure 4). Notably, G-CSF treatment nearly doubled the frequency of Sca-1 expressing BM-derived GFP^+^ cells (2.1 ± 0.6% versus 0.9 ± 0.5%). Similarly, G-CSF statistically increased the Sca-1^+^/GFP^+^ cells, through immunofluorescence staining compared with Saline group (4 w 7.5 ± 1.3 cells/HPF versus 3.5 ± 0.6 cells/HPF, and 8 w 9.4 ± 1.4 cells/HPF versus 4.8 ± 1.0 cells/HPF, *P <* 0.05, Figure 2B). Although G-CSF also promoted GFP^+^ BM stem cells differentiation into c-Kit^+^ renal stem cells, the increased extent was lower than Sca-1^+^ GFP^+^ cells. (Figures 2B-C, 4).

### BM-derived stem cells mediated protective effects for renal ischemia-injury

**Figure 5:**
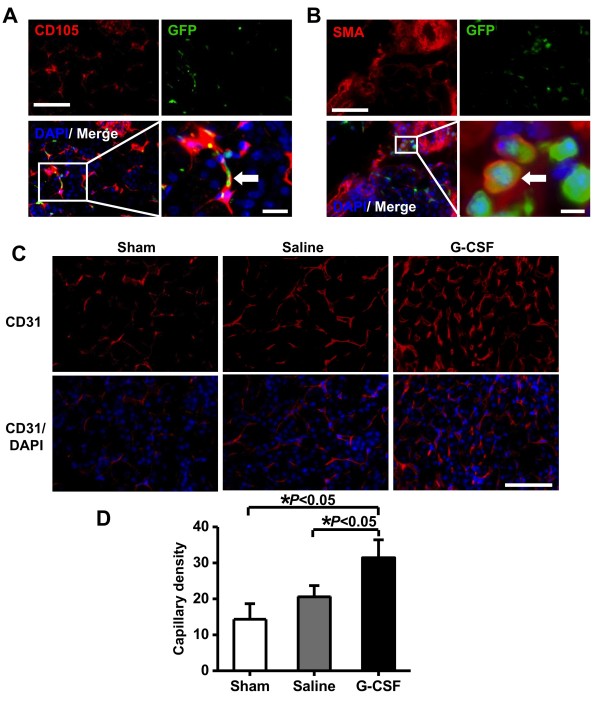
**Analysis of neovascularization.** (**A**) Agiogenesis of BM-derived CD105^+^ endothelial cells and confirmed by GFP co-expression (arrowhead). Scale bars = 50 μm (upward) and 20 μm (below), respectively. (**B**) Immunostaining of α-SMA and GFP demonstrating functional vessels formation of BM derived cells (arrowhead). Nuclear was stained with DAPI. Scale bars = 50 μm (upward) and 5 μm (below), respectively. (**C & D**) Quantitative analysis revealed G-CSF administration can increase capillary density significantly comparing to sham and saline groups. Nuclear was stained with DAPI. Scale bar = 50 μm.

**Figure 6:**
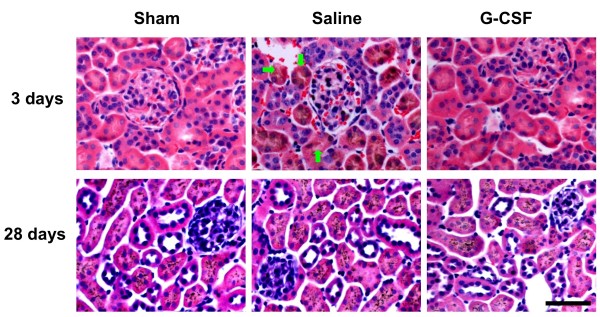
**G-CSF administration can improve kidney recovery from I/R injury.** Representative H&E staining of kidney sections at day 3 and day 28 after I/R injury. At day 3, tubular swelling, necrosis can be detected in saline-treated group (green arrow), but minor damage in G-CSF group. No renal damage could be observed in sham-operated group. At day 28, there are no significant differences among saline, sham, G-CSF groups. Scale bar =50 μm.

To study the profitably angiogenic and/or vasculogenic effects of BM-derived stem cells at week 4 after ischemia, the renal sections were examined by immunofluorescence. CD105, one marker of endothelial cells, was found to be co-expressed in some subset of GFP^+^ cells (Figure 5A). Sections were also stained with smooth muscle marker α-SMA, a part of GFP co-expressing cells showed good match with α-SMA-positive cells in post-ischemic kidneys (Figure 5B). Besides, as shown in Figure 5C, anti-CD31 staining revealed higher microvessels density (MVD) in the G-CSF treated group comparing to the control group (31.4 ± 4.9 capillaries/HPF versus 20.5 ± 3.1 capillaries/HPF, *P <* 0.05, Figure 5C, D). We have also assessed the histological changes in kidney by H&E staining of sham-operated, saline-treated, and G-CSF-treated groups in short and long term following I/R injury (Figure 6). At day 3, there was extensive tubular swelling, necrosis in saline-treated group. In contrast, kidneys from G-CSF treated group showed slight tubular swelling after acute injury, but no renal damage was observed in sham-operated group. By day 28, necrotic injury almost disappeared and regenerating cells were observed to cover the tubules in both saline- and G-CSF-treated groups.

## Discussion

Although therapeutic administration in animal models of acute renal injury suggested that BM stem cells based therapy may improve the recovery of both glomerular and tubular compartments, the suitability of these cell tenants for their new home in kidney has not been well investigated so far. In this study, our results indicate that transplanted BM derived stem cells can engraft, survive in kidney with ischemia injury, and acquire the renal stem cells phenotypes restoring renal ischemia injury. Our studies revealed here for the first time that BM derived stem cells could differentiate into renal stem cells and integrate with host renal cells. Furthermore, this process can be enhanced by G-CSF administration.

Recent works have documented the presence of a reservoir of stem and progenitor cells in the interstitium, papilla, urinary pole of kidney and these cells have been successfully isolated and expanded *ex vivo *[3-10]. The surface markers, such as CD133, CD24, PAX-2, c-Kit and Sca-1, were exploited to define putative renal stem/progenitor cells in kidney [5,6]. These cells, lacked hematopoietic markers, demonstrated self-renewal under culture conditions, and differentiated into epithelial and endothelial cell types *in vitro* and incorporated into tubules following glycerol induced tubular necrosis *in vivo *[6,8]. Several works using renal stem cells also have demonstrated significant improvement in renal function after renal progenitor cells injection [4,16,17]. However, they more focus on the identification of stem cells, and reckon without the isolation efficacy of this kind of cells. The demand for renal stem cells is increasing for end-stage renal disease (ESRD) due to severe shortage of donor organs. Furthermore, as end stage of CKD, kidneys of ESRD are already small and fibrotic, endogenous renal stem cells have exhausted and other origins should be investigated.

The bone marrow constitutes the main reservoir of stem cells, and these cells can egress from the marrow niches, enter the systemic circulation [14]. The role of BM derived stem cells in repair or rejuvenation of tissues and organs that undergo injuries or degeneration has been drawn increasing attention. It was previously proposed that bone marrow derived cells could differentiate into renal cells and contribute to kidney regeneration following renal ischemia/reperfusion injury [15,18]. However, subsequent studies have demonstrated that only a few bone marrow derived cells engraft injured tubules and that their overall contribution to renal repair was negligible [19-21]. Recent results showed that bone marrow derived cells could migrate to damaged kidneys and participate in functional and structural recovery [22-24]. Furthermore, several studies demonstrated that the infusion of human or mice derived MSCs contribute to the kidney regeneration following AKI [25,26]. Thus blood-borne renal cells may be detected exclusively when the peripheral blood contains a large number of transplanted BM derived stem cells in long term. In this study, we set up BMT model other than bone marrow cells injection directly into kidney. The transplanted GFP^+^ stem cells will migrate via the bloodstream throughout all experimental courses for several months, which would increase the possibility of BM derived stem cells residing in kidney and the usage of G-CSF would mobilize more HSCs into circulation. We confirmed that BM cells can integrate into damaged kidney. Moreover, we found for the first time that BM cells can differentiate into Sca-1^+^/CD45^-^, or c-Kit^+^/CD45^-^ renal stem cells over one to two months.

The efficiency of BM cell-based therapy to augment recovery from damaged tissues depends on not only efficient delivery of these cells to the desired target tissue, but also sufficient amount of stem cells. HSCs are unique in their ability to migrate to various sites, ensuring the safety and integrity of their regenerative potential. However, the number of traffic HSCs in blood stream is at an extremely low level. G-CSF has been used for the collection of BM cells used in allogeneic or syngeneic stem cell transplantation [27,28]. After G-CSF mobilization, the HSCs can release from their storage niche into circulation, ending their journey in the injured organs. We confirmed that G-CSF-treatment would mobilize BM stem cells from the bone marrow into ischemic kidneys and increase BM-derived renal stem cells after acute kidney ischemia, which perhaps is one of mechanisms of damaged kidney repair given by G-CSF. Previous reports showed that G-CSF stimulates angiogenesis in infracted kidneys, which acts to promote functional recovery of the damaged tissue [2,28,29]. These observations are based on the fact that HSCs mobilized by G-CSF can differentiate into vascular endothelial cells. A secondary finding in their studies, which was not explored further, was the beneficial effects of angiogenesis. Through increasing the vessel density, raising blood flow, G-CSF may enhance more BM-derived stem cells cycling and storing in the ischemic kidneys, and then the environment of injured kidneys may induce BM-derived cells incorporating into renal cells, getting the phenotypes of renal progenitors, differentiating into renal stem cells and terminal cells, finally participating in kidney functional repair. In addition, BM derived stem cells formed nested capillaries that might be helpful in supplying more oxygen and providing secondary protection of tubular cells [5]. Further studies are required to provide insight into the mechanisms.

## Conclusion

In conclusion, the results of the present study confirmed that bone marrow derived stem cells could differentiate into renal stem cells. Furthermore, G-CSF enhanced recruitment of BM-derived renal progenitor cells and neovascularization in a murine AKI model to promote repair. The result of this study opens a new perspective for bone marrow therapy in kidney injury.

## Competing interests

The authors declare that they have no competing interests.

## Authors’ contributions

ZL, YX and DK were the principal investigators and take primary responsibility for the paper. ZL, DK, YX, HL, XJ, GF, ZH, YX and XX conceived and designed the experiments. XX, GF, YC, QZ, and ZL performed the experiments. XX participated in the statistical analysis. XJ, DK and ZL wrote the paper. All authors read and approved the final manuscript.

## Pre-publication history

The pre-publication history for this paper can be accessed here:

http://www.biomedcentral.com/1471-2369/13/105/prepub

## Supplementary Material

Additional file 1: Figure S1 Immunohistochemical staining of GFP. Six months after I/R injury, consecutive sections of kidney were prepared and stained with GFP antibody or PBS (as control) and subsequently diaminobenzidine (DAB). GFP expression is observed in the glomeruli and interstitium. Arrows in (**D**) indicate the corresponding glomeruli in (**A**). Abbreviation: G, glomerulus; GFP, green fluorescent protein; P, renal pelvis; V, vessel. Original magnification: ×50 (**A, D**) , ×100 (**B, E**), ×200 (**C, F**). Click here for file
