# Supplementary figures and images for: Bone marrow-derived cells can acquire renal stem cells properties and ameliorate ischemia-reperfusion induced acute renal injury
Source: BMC Nephrol. 2012 Sep 10;13:105. doi: 10.1186/1471-2369-13-105 (PMC3505151; doi:10.1186/1471-2369-13-105)

## Slide 1
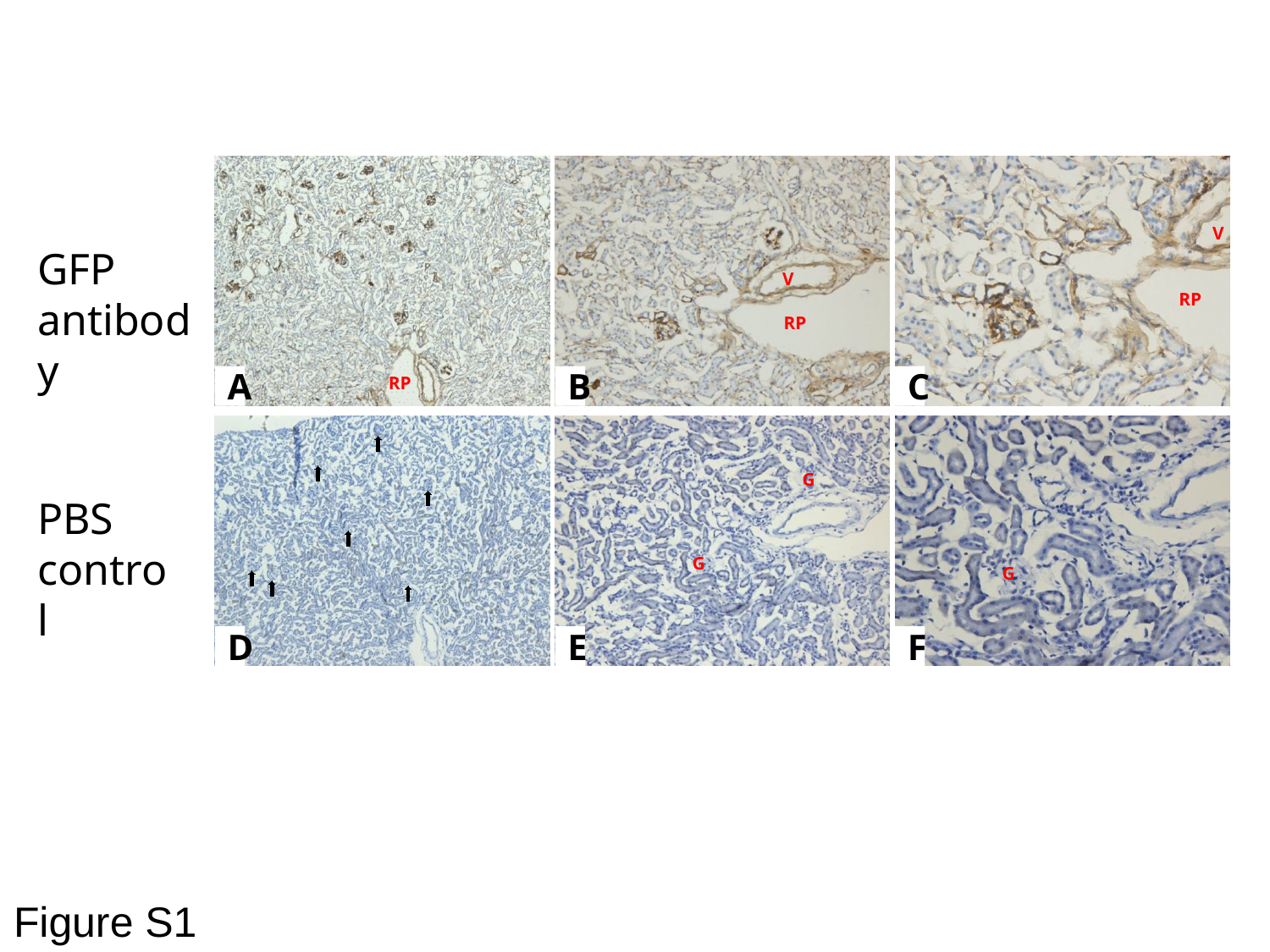

A
RP
B
V
RP
C
V
RP
GFP antibody
D
E
G
G
F
G
PBS control
Figure S1

Supplement: Additional file 1: Figure S1 — Immunohistochemical staining of GFP. Six months after I/R injury, consecutive sections of kidney were prepared and stained with GFP antibody or PBS (as control) and subsequently diaminobenzidine (DAB). GFP expression is observed in the glomeruli and interstitium. Arrows in (D) indicate the corresponding glomeruli in (A). Abbreviation: G, glomerulus; GFP, green fluorescent protein; P, renal pelvis; V, vessel. Original magnification: ×50 (A, D) , ×100 (B, E), ×200 (C, F). [file 1471-2369-13-105-S1.pptx]
